# Apathy in Patients with Parkinson's Disease Correlates with Alteration of Left Fronto-Polar Electroencephalographic Connectivity

**DOI:** 10.3389/fnagi.2017.00262

**Published:** 2017-08-15

**Authors:** Florian Hatz, Antonia Meyer, Ronan Zimmermann, Ute Gschwandtner, Peter Fuhr

**Affiliations:** Department of Neurology, Hospitals of University of Basel Basel, Switzerland

**Keywords:** Parkinson's disease, apathy, executive functions, quantitative electroencephalography, neuropsychology

## Abstract

**Introduction:** Quantitative electroencephalography (QEEG) brain frequency and network analyses are known to differentiate between disease stages in Parkinson's disease (PD) and are possible biomarkers. They correlate with cognitive decline. Little is known about changes in brain networks in relation to apathy.

**Objective/Aims:** To analyze changes in brain network connectivities related to apathy.

**Methods:** 40 PD patients (14 PD with mild cognitive deficits and 26 PD with normal cognition) were included. All patients had extensive neuropsychological testing; apathy was evaluated using the apathy evaluation score (AES, median 24.5, range 18–39). Resting state EEG was recorded with 256 electrodes and analyzed using fully automated Matlab® code (TAPEEG). For estimation of the connectivities between brain regions, PLI (phase lag index) was used, enhanced by a microstates segmentation.

**Results:** After correction for multiple comparisons, significant correlations were found for single alpha2-band connectivities with the AES (*p*-values < 0.05). Lower connectivities, mainly involving the left fronto-polar region, were related to higher apathy scores.

**Conclusions:** In our sample of patients with PD, apathy correlates with a network alteration mainly involving the left fronto-polar region. This might be due to dysfunction of the cortico-basal loop, modulating motivation.

## Introduction

Apathy is beside depression one of the most common non-motor, neuropsychiatric symptoms in patients with Parkinson's disease (PD) (Pedersen et al., 2010; Cubo et al., 2012). Apathy is defined as a primary loss of motivation, a loss of interest and effortful behavior (Levy and Dubois, 2006; Starkstein et al., 2012) and has significant impact on quality of life (Chaudhuri et al., 2011). Flexibility in shifting between behaviors and the ability to bypass distractions are required for goal-directed actions and dysfunction of these abilities may play an important role in development of apathy (Goschke and Bolte, 2014). Alterations of these executive functions are highly prevalent in PD and correlate with apathy (Dujardin et al., 2009; Meyer et al., 2015). Executive functions can be divided into initiation, inhibition and shifting (Drechsler, 2007). Among these, only initiation is associated with beginning apathy (Meyer et al., 2015). Initiation is defined as the ability to start intentional, self-motivated actions. Impairments in this process are associated with tests, in which patients are self-generating a processing speed or starting an action. In the same study, mild apathy was shown not to be associated with depression.

Levy and Dubois differentiated 3 subtypes of disrupted processing in the prefrontal-basal ganglia system related to apathy (Levy and Dubois, 2006): emotional-affective processing, cognitive processing and auto-activation processing. In conventional neuropsychological test settings, executive dysfunctions explain mainly deficits in cognitive processing related to apathy. This setting is less sensitive for deficits in emotional-affective and auto-activation processing inspite of their clinical importance for behavior of patients with PD suffering from anhedonia (Giovannoni et al., 2000). Interestingly, however, anhedonia is not associated with apathy (Isella et al., 2003).

In neuroimaging studies apathy is associated to the lateral prefrontal cortex, the anterior cingulate cortex, insula and the basal ganglia, explicitly the medial superior frontal gyrus (Klaasen et al., 2017). An analysis of functional connectivity using quantitative imaging technique showed a reduction of functional connectivity in left sided limbic, striatal and frontal regions (Baggio et al., 2015). These observations are in line with the concept of Miller et al. (Miller and Cohen, 2001), assigning an important role to the prefrontal cortex in cognitive control.

Results of quantitative analysis of electroencephalography (QEEG), including frequency, connectivity and brain network analysis, are correlating to the disease stage and the cognitive decline, related to PD dementia (Olde Dubbelink et al., 2014a,b). However, little is known about alterations in QEEG analysis, related to apathy and executive dysfunctions.

The present study aims at analyzing changes in brain network connectivities related to apathy. Based on literature, PD patients with apathy are expected to have lower connectivity in the left frontal regions. For estimation of the connectivity between brain regions the phase lag index (PLI) was used, a connectivity measure little influenced by effects of volume conduction (Stam et al., 2007). PLI has been shown to be a reliable measure over time (Hardmeier et al., 2014). PLI calculations were combined with a microstates segmentation, as this combined method shows a still higher test-retest reliability compared with the conventional PLI calculations (Hatz et al., 2016).

## Methods

### Patients

Fifty-eight patients with PD were recruited between October 2011 and April 2013 from the movement disorders clinic of Hospital of the University of Basel or through advertisements. The patients were participants of a Cognitive training-study (Zimmermann et al., 2014) and underwent neuropsychological, psychiatric and neurological assessment. Inclusion criteria for the study were idiopathic PD according to UK Parkinson‘s Disease Brain Bank Criteria (Gibb and Lees, 1988) and written informed consent. Patients were excluded if they had moderate or severe dementia (MMSE ≤ 24; Folstein et al., 1975), insufficient knowledge of the german language, other severe brain disorders, alcohol or drug dependency. For the calculations of this study, the data of *n* = 40 (14 PD with mild cognitive deficits and 26 PD with normal cognition) has been analyzed. All patients were on dopaminergic medication and were tested while they were in the ON state.

Apathy was measured with the German version of the Apathy Evaluation Scale (AES) (Lueken et al., 2006) filled out by a relative or a person close to the patients. Symptoms of depression were measured with a self-rating scale, the German version of Beck Depression Inventory II (BDI) (Beck et al., 1961). The severity of motor signs was assessed using Unified Parkinson‘s Disease Rating Scale (UPDRS) (Fahn and Elton, 1987) subscale III, applied by trained neurologists.

### Neuropsychology

Patients were examined with a comprehensive battery of neuropsychological tests (Zimmermann et al., 2014). Raw scores of tests were transformed *z*-values. According to Table 1 a sum score for initiation was calculated.

**Table 1:** Performance in tests measuring initiation (executive function).

	**Test**	**Variable**	**Mean (SD)**	**Median**	
Initiation^a^	Phonemic fluency	correct answers	19.77 (4.67)	21	Morris et al., 1989
	Semantic fluency	correct answers	23.78 (7.30)	22.5	Thurstone and Thurstone, 1948
	5 Point Test	correct figures	46.48 (15.82)	43.5	Regard et al., 1982
	TMT	TMT A	15.82 (3.66)	15	Reitan, 1955
	Stroop	naming colors	1.92 (2.25)	1.5	Stroop, 1935

a *Classification according to Drechsler (2007)*.

*All values represent z-transformed values. TMT = Trail Making Test*.

*Cronbach‘s α for initiation: 0.79*.

### EEG-recording

EEG was recorded with a 256-channel EEG System (Netstation 300, EGI Inc. Eugene, OR 97403, USA; DC-amplifier; sampling rate: 1,000 Hz; high pass filter: 0.01 Hz; vertex-reference, impedance ≤ 40 kΩ). Subjects were instructed to relax, but to stay awake and to minimize eye and body movements. A continuous EEG with closed eyes was recorded for 12 min. During data acquisition, a subset of electrodes was monitored online by a technician to check for vigilance and artifacts.

### Processing of EEG data

**Figure 1:**
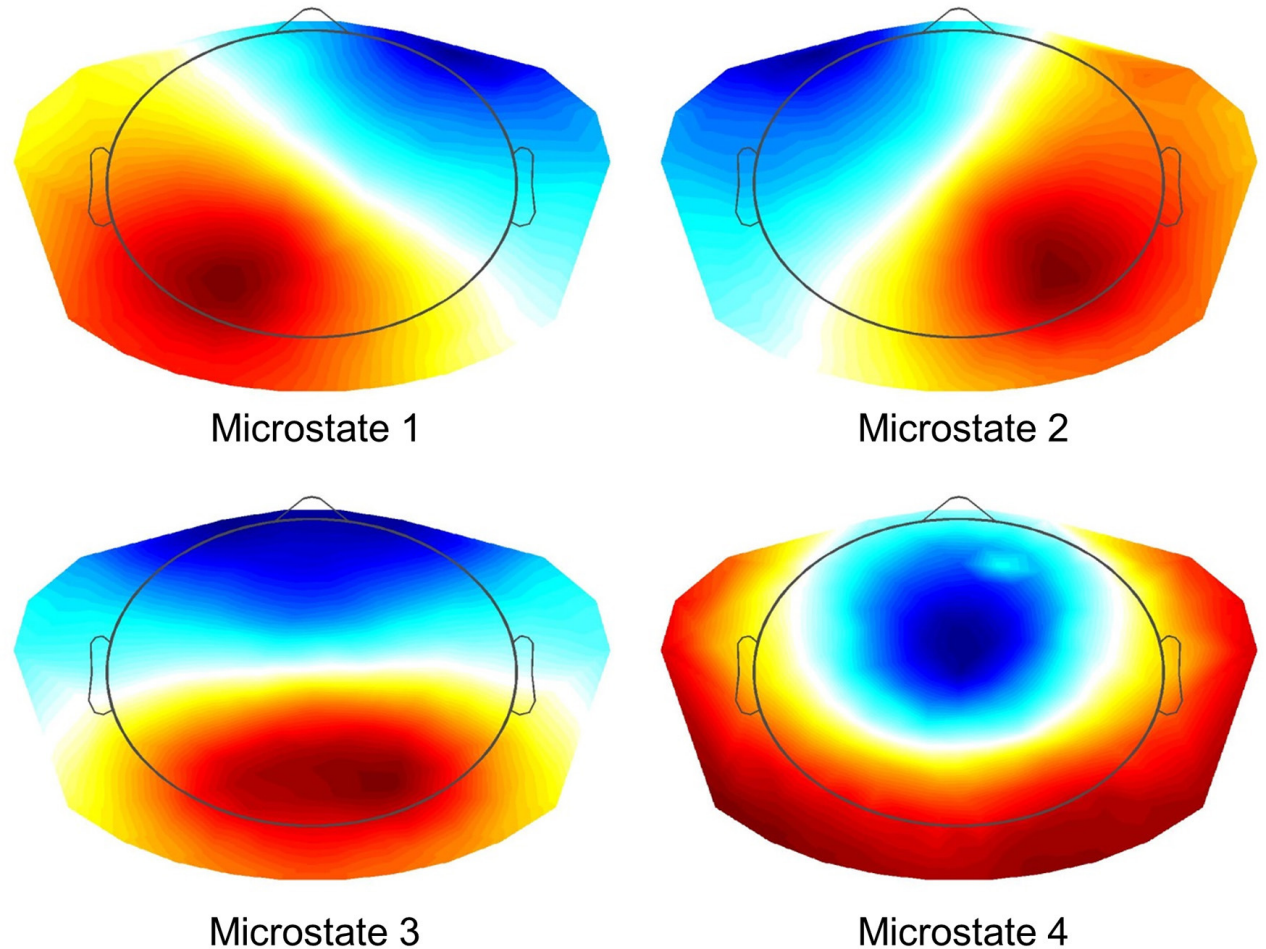
Microstates (K-means Clustering). Resulting four microstates after K-means clustering and selecting optimal number using L-curve via an adaptive pruning algorithm on the Krzanovski-Lai criterion (Krzanowski and Lai, 1988).

All EEG data were automatically preprocessed using TAPEEG, as described in a previous publication (Hatz et al., 2015). In brief, PLI measures were calculated using a Butterworth filter to four predefined frequency bands (theta: 4–8 Hz, alpha1: 8–10 Hz, alpha2: 10–13 Hz and beta: 13–30 Hz) and calculated as described by Stam et al. (2007). PLI calculation was combined with microstates segmentation (msPLI) (Hatz et al., 2016). In a previous publication msPLI showed an increased test-retest-reliability of time in healthy subjects compared to classical PLI (Hatz et al., 2016). For calculation of msPLI the raw EEG were segmented into four microstates (Figure 1) and the Hilbert-transformation was applied to the full-length EEG using a sliding window of 4 s with a 50% Hanning-window. For every microstate class four stitched periods of each 4,000 phase differences (= 4 s) were then extracted, using the time frames indicated by the microstate label vector (Supplementary Figure 1). The number of 4 epochs per microstate, subject and frequency band was selected, as this minimal amount of epochs per microstate was available in almost all EEG given the recording time of EEG data. The 16 resulting matrices (four epochs × four microstates) were averaged per subject. For statistical analysis, electrodes were grouped into 22 regions of interest (ROI) (Supplementary Figure 2), 11 regions per hemisphere excluding electrodes in the midline, neck and face. Graph analysis results were calculated according to Table 2.

**Table 2:** Graph measures as calculated for each subject.

**Name**		**Formula**	**References**
Degree		=mean(W) (all links of a single node)	
Average clustering coefficient	Cw	*CW* = *mean*(*c*)	Stam et al., 2009
		$C_{i}=\frac{\begin{array}{c} \sum_{k\neq i}\sum_{l\neq i}W_{ik}W_{il}W_{kl}\\ l\neq k \end{array}}{\begin{array}{c} \sum_{k\neq i}\sum_{l\neq i}W_{ik}W_{il}\\ l\neq k \end{array}}$	
Normalized clustering coefficient	Gamma	Cw / Cr	Spoormaker et al., 2010
		(Cr = average of 50 randomized input matrices)	
Average path length	Lw	*L* = 1/*W*	Stam et al., 2009
		*L* = ∞ (*if w* = 0)	
		$Lw=\frac{1}{\frac{1}{N\left(N-1\right)}*\sum_{i=1}^{N}\sum_{j\neq i}^{N}\left(1/L_{ij}\right)}$	
Normalized average path length	Lambda	Lw / Lr	Spoormaker et al., 2010
		(Lr = average of 50 randomized matrices)	
Degree correlation	Rw	Rw = Pearson correlation of degrees of pairs of neighbors	Newman, 2003
Degree diversity	Kw	$Kw=\frac{\langle degree^{2}\rangle }{\langle degree\rangle }$	Stam and van Straaten, 2012
Radius	Radius	=min (L)	Wang et al., 2008
Diameter	Diameter	=max(L)	Wang et al., 2008

*W = PLI of single link*.

### Statistics

The apathy total score was not normally distributed (KSLT: Kolmogorov Smirnow Lilliefors Test; *p* < 0.01) therefore, this variable was square root transformed, leading to a non-significant *p*-value (*p* = 0.34) of the KSLT. After z-score transformation, the executive function variables were averaged; variables indicating good performance in smaller values were reversed (Table 1). To check the validity of the subprocesses of initiation, the intercorrelations between all the collected z-transformed cognitive variables and internal consistency (Cronbach‘s alpha) were calculated. As defined by George et al. (2002), acceptable internal consistency was set at Cronbach‘s α of greater or equal to 0.70.

As the results of connectivity analysis were not normally distributed, resulting msPLI values were log transformed.

Spearman-rank was used to estimate the correlation of connectivity values to neuropsychiatric and neuropsychological outcome measures. Age, gender, education, MMS and LED were used as confounding variables. To correct for those variables for every single connectivity value, a linear regression was calculated using the variables to predict the connectivity value. The residuums of the connectivity values were used for the correlation analyses. To correct for multiple comparisons permutation tests (10'000 permutations) were used (Nichols and Holmes, 2002). Results with *p*-values < 0.05 were considered significant. Analyses were done using TAPEEG (Hatz et al., 2015).

## Results

### Demographics and neuropsychology

The summary of demographics and neuropsychology is represented in Table 3.

**Table 3:** Demographic features of 40 patients with Parkinson's disease.

	**Mean (Std)**	**Median (Quartiles)**
Age	68.0 (7.7)	68.5 (62–73)
Sex	35% female	
Education (years)	14.6 (3.0)	14.5 (12–16)
Levodopa-Equivalence-Dose	623.1 (422.7)	569.3 (255–777)
UPDRS III	15.4 (11.2)	13.0 (5–21)
AES	26.2 (6.2)	24.5 (20–30)
BDI (Beck Depression Inv.)	7.4 (4.3)	7.0 (3–11)
MMS	28.8 (1.0)	29.0 (28–30)
Initiation (Sum score)	13.05	(5.07) std = 0.72

### Quantitative EEG

**Figure 2:**
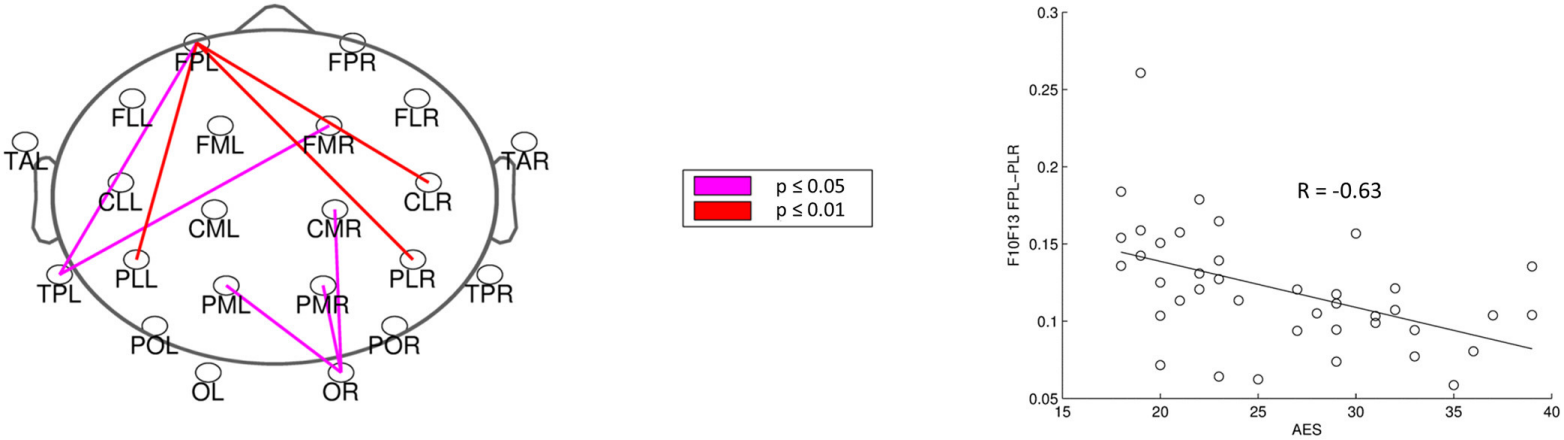
Correlation of AES raw scores and alpha2-connectivity. (*p*-value < 0.05 = violett; < 0.01 = red).

**Figure 3:**
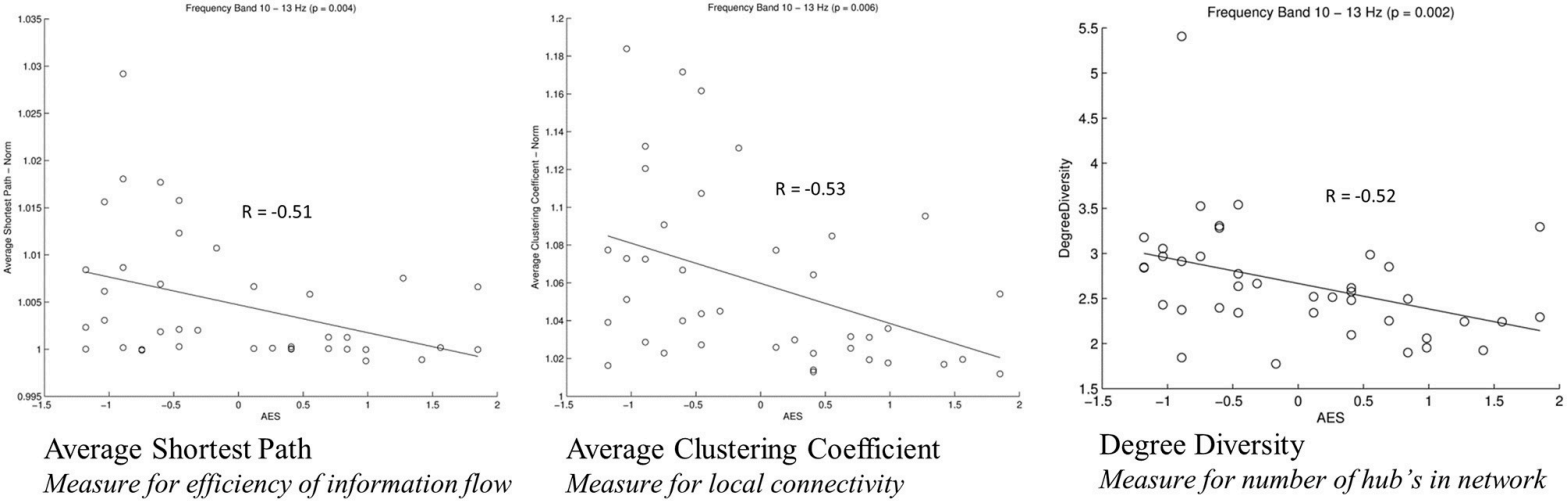
Correlation of graph measures and alpha2-connectivity. Average Shortest Path (measure for efficiency of information flow); Average Clustering Coefficient (Measure for local connectivity); Degree Diversity (Measure for number of hub's in network).

**Figure 4:**
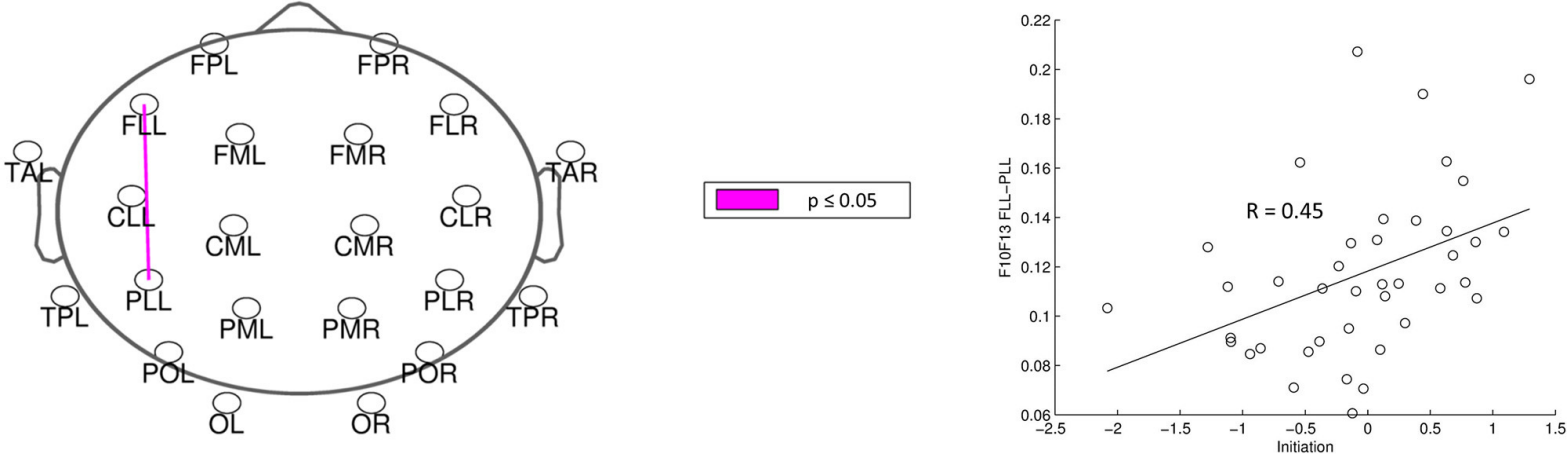
Correlation of initiation and alpha2-connectivity. (*p-value* < 0.05 = violett).

**Figure 5:**
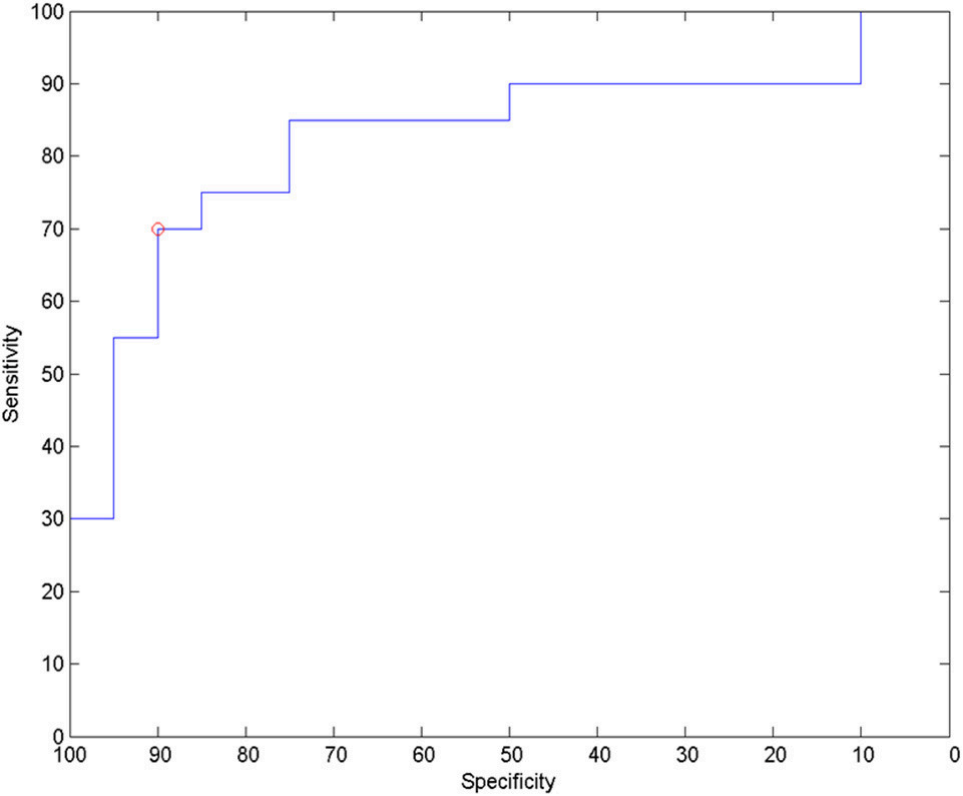
ROC for classification to low apathy (AES ≤ 24) and mild apathy (AES ≥ 25) using alpha2-band msPLI_alpha2 (left frontopolar—right parietal lateral).

For analysis of signal-space frequency results, a borderline significant correlation of AES and relative delta bandpower in the right frontal region was found, no correlation to the other frequency bands (Table 4). For connectivity analysis, significant results were found for alpha2-band results only. After correction for multiple comparisons, significant correlations with the AES were found for alpha2-band connectivities involving the left fronto-polar region (*p*-values ≤ 0.05). Lower connectivities were related to higher apathy scores (Figure 2). Graph measures of alpha2-band networks correlated to AES scores (Figure 3). Analysis of initiation showed only a barely significant correlation to alpha2-band connectivity fronto-parietal left (Figure 4). Using a median-split for the AES results (median = 24.5) and the results for alpha2-msPLI-connectivity at FPL-PLR, a classification of low vs. mildly apathetic PD-patients is possible with a sensitivity of 70%, a specificity of 90% and a AUC of 82.5 (Figure 5).

**Table 4:** Results: Spearman's correlation of AES and frequency bands.

	**Delta**		**Theta**		**Alpha1**		**Alpha2**		**Beta**	
	***R***	***p*-value**	***R***	***p*-value**	***R***	***p*-value**	***R***	***p*-value**	***R***	***p*-value**
Frontal_L	0.35	0.22	0.12	0.80	−0.15	0.85	−0.27	0.26	−0.24	0.51
Frontal_R	0.47	***0.05***	0.16	0.64	−0.19	0.70	−0.31	0.17	−0.30	0.27
Central_L	0.36	0.16	0.16	0.67	−0.13	0.92	−0.26	0.30	−0.22	0.56
Central_R	0.34	0.21	0.16	0.63	−0.08	0.98	−0.26	0.28	−0.25	0.41
Temporal_L	0.28	0.51	0.15	0.67	−0.15	0.71	−0.31	0.18	−0.31	0.25
Temporal_R	0.27	0.43	0.19	0.52	−0.10	0.77	−0.32	0.14	−0.35	0.15
Parietal_L	0.27	0.43	0.17	0.60	−0.13	0.92	−0.27	0.26	−0.22	0.56
Parietal_R	0.36	0.16	0.19	0.53	−0.17	0.81	−0.30	0.19	−0.17	0.71
Occipital_L	0.36	0.20	0.12	0.80	−0.17	0.81	−0.32	0.13	−0.21	0.59
Occipital_R	0.42	0.07	0.18	0.57	−0.21	0.60	−0.34	0.11	−0.15	0.83

*Frequency results were calculated for 22 regions and correlated to AES. For presentation results of R and p-values were averaged to 10 regions. p-values ≤ 0.05 are represented in bold*.

## Discussion

In our sample of patients with PD, apathy correlates with network alterations in the alpha2 band mainly involving the left fronto-polar region. The effect is visible even though patients in our study sample were only slightly affected by apathy, and might be due to dysfunction of the cortico-basal loop, modulating motivation (Levy and Dubois, 2006). A dysfunction of these cortico-basal loops is also linked to executive functions (Alvarez and Emory, 2006). According to Drechsler (2007) executive functions can be subdivided in initiation, shifting and inhibition. As a previous work of our group showed only initiation to be linked to apathy scores (Meyer et al., 2015), only the sum score for initiation was included in the present analysis. This sum score was only weakly correlated to the alpha2-band connectivity. However, the correlated connectivity result was located fronto-parietal left-sided, in nearly the same region as the connectivities correlated to apathy. Furthermore, PD patients with apathy had lower average clustering coefficient (Gamma), average path length (Lambda) and degree diversity (Kw). Gamma represents the amount of local connectivity, Lamba the efficiency of information flow and degree diversity the scale free distribution. Higher scale free distribution is linked to the amount of hubs in a network (de Haan et al., 2012). Stam et al. described 2012 a model of physiological brain networks, showing the ‘normal brain’ as an intermediate network state between a regular, a random and a scale-free network. Reduction of Gamma, Lambda, and Kw would indicate mainly a shift toward a more random network state with reduced local connectivity, reduced number of hubs and possibly slightly better efficiency of information flow on the global scale.

Only the alpha2 band shows significant results. Increase of alpha2 power has been linked to an increased capacity to initiate new tasks (Klimesch, 1999), strongly related to the concept of initiation as one of the executive functions (Drechsler, 2007). The observed reduction of alpha2-band connectivity in the left fronto-polar region is in accordance with the concept of Miller et al. (Miller and Cohen, 2001), allocating to the prefrontal cortex an important role for cognitive control, and the reduction of connectivity might represent a reduction of the capability to initiate a new task, leading to apathy.

As a limitation of the present study, all patients included in the study were only mildly affected by apathy. Therefore confirmation of results in a group of PD patients with in median higher apathy scores is warranted.

## Ethics statements

Written informed consent was given by all study participants. The study was approved by the local ethics committee (Ethikkommission beider Basel, Nr. 135/11).

## Author contributions

FH helped conducting the study, processed the EEG data and drafted the manuscript. AM and ZR helped conducting the study. PF and UG conceived the study, participated in its design and coordination and helped to draft the manuscript. All authors read and approved the final manuscript.

### Conflict of interest statement

UG: Support of research from Mach-Gaensslen-Foundation, Gossweiler Foundation, Parkinson Schweiz, Synapsis Foundation, Botnar Foundation, PF: Support of research from Swiss National Science Foundation, Mach-Gaensslen-Foundation, Gossweiler Foundation, Parkinson Schweiz, Synapsis Foundation, Botnar Foundation, Freiwillige Akademische Gesellschaft Basel, Novartis Research Foundation, Novartis, Roche, AbbVie, Hedwig-Widmer-Stiftung. The other authors declare that the research was conducted in the absence of any commercial or financial relationships that could be construed as a potential conflict of interest.

## Supplementary material

Supplementary Figure 1 Calculation of msPLI. After phase transformation of signal A and B using Hilbert-transformation, the phase-difference over time is calculated, and the resulting vector segmented according to the previously calculated microstates. PLI values are calculated based on the resulting four different segments.

Supplementary Figure 2 Twenty-two regions for connectivity analysis.
