# Supplementary figures and images for: Apathy in Patients with Parkinson's Disease Correlates with Alteration of Left Fronto-Polar Electroencephalographic Connectivity
Source: Front Aging Neurosci. 2017 Aug 15;9:262. doi: 10.3389/fnagi.2017.00262 (PMC5559507; doi:10.3389/fnagi.2017.00262)

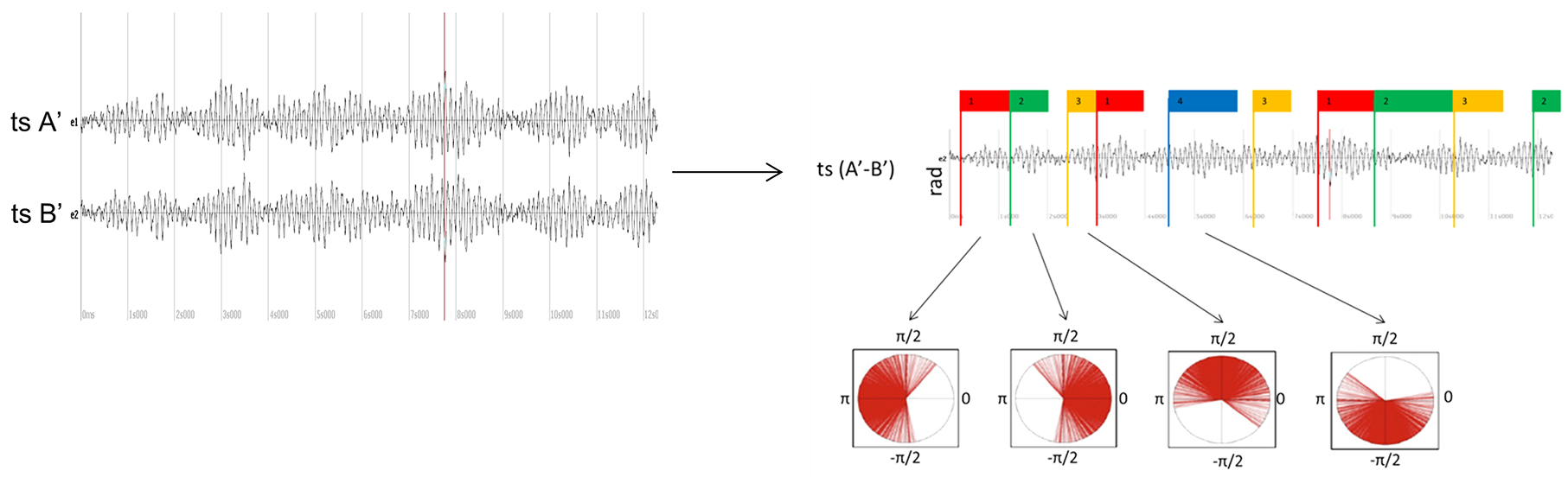

Supplement: Supplementary Figure 1 — Calculation of msPLI. After phase transformation of signal A and B using Hilbert-transformation, the phase-difference over time is calculated, and the resulting vector segmented according to the previously calculated microstates. PLI values are calculated based on the resulting four different segments. [file Image1.TIF]

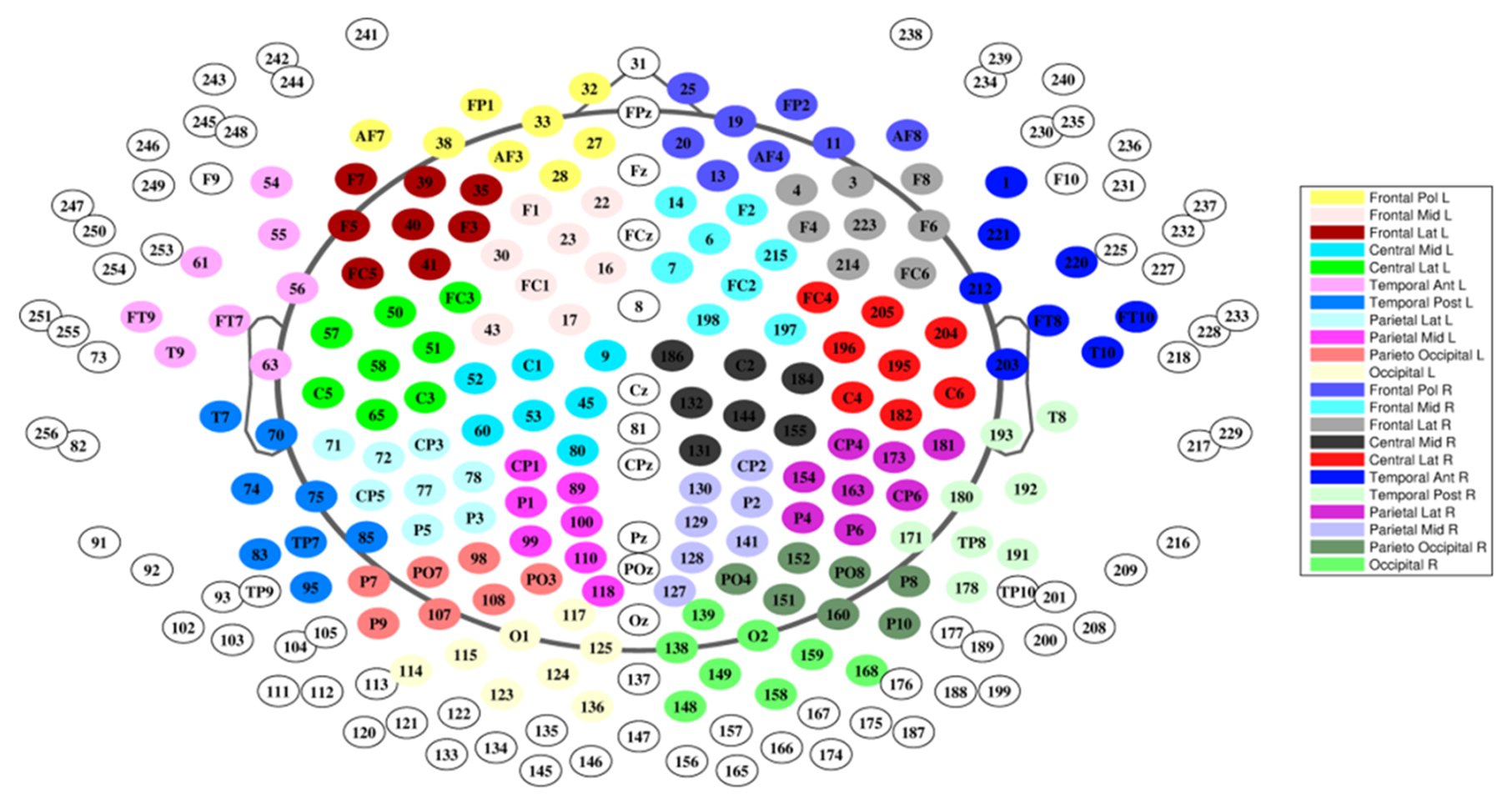

Supplement: Supplementary Figure 2 — Twenty-two regions for connectivity analysis. [file Image2.TIF]
